# PAB-1, a *Caenorhabditis elegans* Poly(A)-Binding Protein, Regulates mRNA Metabolism in germline by Interacting with CGH-1 and CAR-1

**DOI:** 10.1371/journal.pone.0084798

**Published:** 2013-12-19

**Authors:** Sunhee Ko, Ichiro Kawasaki, Yhong-Hee Shim

**Affiliations:** Department of Bioscience and Biotechnology, Institute of KU Biotechnology, Konkuk University, Seoul, South Korea; CNRS UMR7622 & University Paris 6 Pierre-et-Marie-Curie, France

## Abstract

Poly(A)-binding proteins are highly conserved among eukaryotes and regulate stability of mRNA and translation. Among *C. elegans* homologues, *pab-1* mutants showed defects in germline mitotic proliferation. Unlike *pab-1* mutants, *pab-1* RNAi at every larval stage caused arrest of germline development at the following stage, indicating that *pab-1* is required for the entire postembryonic germline development. This idea is supported by the observations that the mRNA level of *pab-1* increased throughout postembryonic development and its protein expression was germline-enriched. PAB-1 localized to P granules and the cytoplasm in the germline. PAB-1 colocalized with CGH-1 and CAR-1 and affected their localization, suggesting that PAB-1 is a component of processing (P)-bodies that interacts with them. The mRNA and protein levels of representative germline genes, *rec-8*, *GLP-1*, *rme-2*, and *msp-152*, were decreased after *pab-1* RNAi. Although the mRNA level of *msp-152* was increased in *cgh-1* mutant, it was also significantly reduced by *pab-1* RNAi. Our results suggest that PAB-1 positively regulates the mRNA levels of germline genes, which is likely facilitated by the interaction of PAB-1 with other P-body components, CGH-1 and CAR-1.

## Introduction

Poly(A)-binding proteins (PABPs) have been identified in many organisms, from yeast, flies, and mice to human [1–4]. PABPs are classified into 2 categories: nuclear PABPs, which are necessary for mRNA maturation; and cytoplasmic PABPs, which are involved in mRNA metabolism [5,6]. PABPs are composed of RNA recognition motifs and a C-terminal domain that is necessary for protein-protein interaction [7]. PABPs influence many aspects of mRNA metabolism, such as transport from the nucleus to the cytoplasm, protection of mRNAs, and translation through the formation of a “closed loop” structure [8–10]. PABPs are also components of the stress granules in which some mRNAs are transiently sequestered during stress in mammals [11]. Stress granules interact with processing (P)-bodies in mammalian cells [12]. P-bodies are cytoplasmic aggregates of RNA and proteins that contain translational repressors and mRNA decay machinery [13,14]. P-bodies are concentrated into foci and some components are diffused throughout the cytoplasm [15]. In *Caenorhabditis elegans*, stress granules and P-bodies are colocalized and a subset of genes that enhances formation of P-bodies also regulates the formation of stress granules [16]. Germ granules are germline-specific cytoplasmic organelles found in the germ cells of many species including *C. elegans*, where they are called as P granules [17]. P granules consist of mRNAs and multiple RNA-binding proteins, some of which were shown to be essential for the germline development. It was also shown that some of *C. elegans* P-body component orthologs, including CGH-1 and CAR-1, colocalize to P granules in the germ cells [15,18–20]. In *C. elegans*, 2 cytoplasmic poly(A)-binding proteins, PAB-1 and PAB-2, and a nuclear poly(A)-binding protein, PABP-2 (also known as PAB-3), have been identified. PAB-1 and PAB-2 are functionally redundant in the soma, but PAB-1 is essential for the germline development [21–23].

*C. elegans* adult hermaphrodite gonads contain mitotic germline stem cells, meiotic germ cells, and differentiating gametes from the distal to proximal orientation [24,25]. In the first larval stage (L1), the gonad primordium contains 2 somatic gonadal precursor cells, Z1 and Z4, and 2 primordial germ cells, Z2 and Z3. The number of germ cells gradually increases during larval development to as many as 1000 germ cells in each of the 2 gonadal arms and they form syncytia in an adult hermaphrodite [24,25]. During the course of development, all the germ cells, except mature sperm, contain P granules in their perinuclear region, which eventually disperse into the cytoplasm during oogenesis [17,26]. In the gonad arm, most germline mRNAs are transcribed primarily in the pachytene stage germ cells and are transported through the nucleus-free core of germline syncytial cytoplasm, called the rachis, to the proximal region; translation occurs in a temporally and spatially modulated fashion depending on the needs of each protein product [27–31]. During these processes, multiple germline-enriched RNA-binding proteins play critical roles [32]. Therefore, elucidating the regulatory mechanisms of mRNA metabolism by these RNA-binding proteins is essential to understand the process of *C. elegans* germline development at the molecular level.

*pab-1* mutants show mitotic proliferation defects in the germline [21–23]. Nevertheless, it has not been clear whether *pab-1*, a seemingly general mRNA regulator, has a specific function only for mitotic proliferation; or *pab-1* also has essential functions in later stages of germline development, but those phenotypes are masked by the proliferation defects. In this study, we examined possible functions of *pab-1* in the later stages of germline development by treating synchronized worms with *pab-1* RNAi at each larval stage. We also examined possible functional association of PAB-1 with other RNA-binding proteins. Here, we show that PAB-1 colocalizes and interacts with P-body components, CGH-1 and CAR-1, and promotes the entire processes of postembryonic germline development by maintaining the mRNA levels of germline genes. An earlier study demonstrated that PAB-1 is actively involved in translation of germline proteins through its cosedimentation with polysomes [33]. Our data obtained in this study reveal that PAB-1 is also involved in mRNA metabolism through its association with P-body components.

## Results

### *pab-1* is required throughout germline development

**Figure 1:**
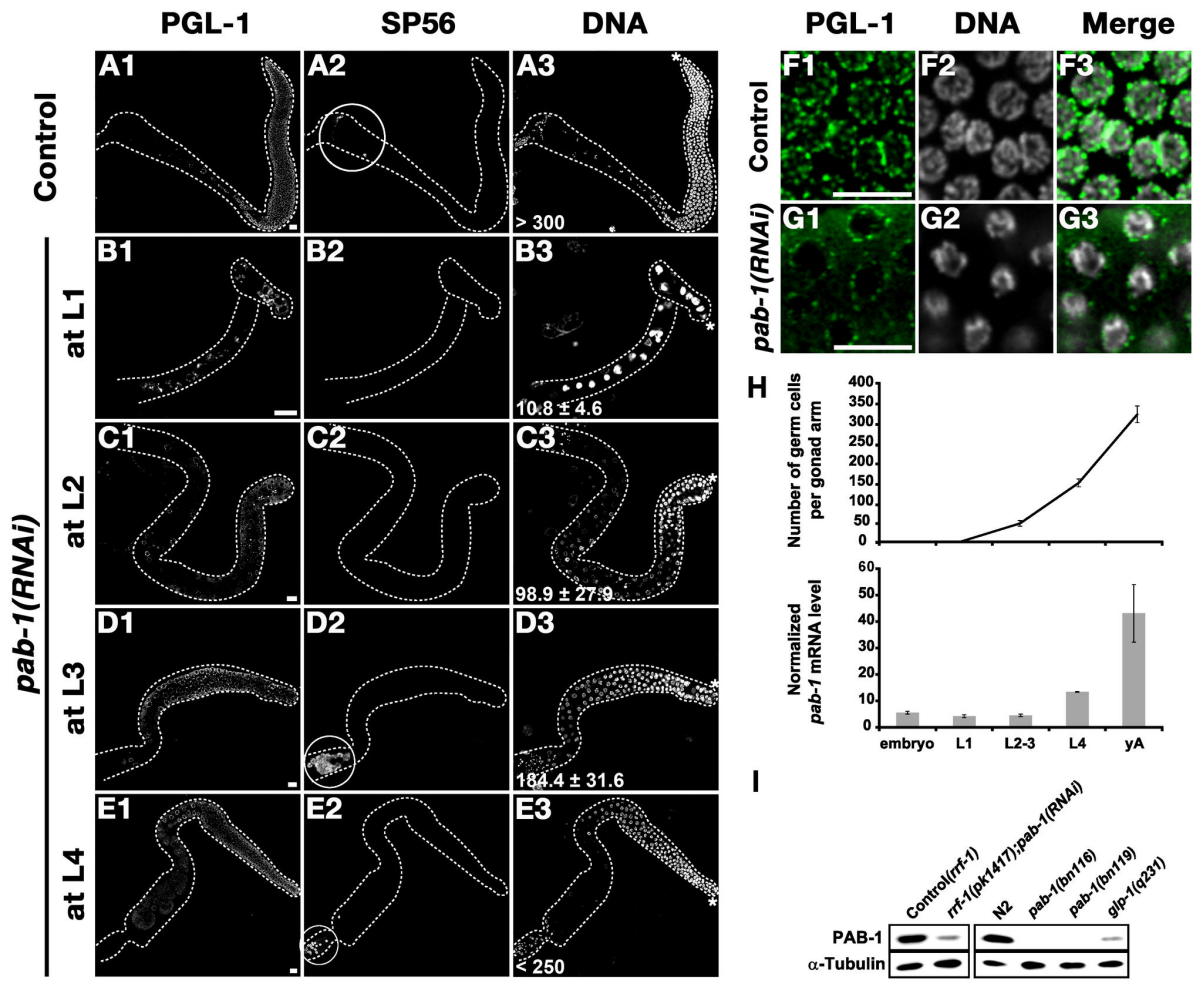
*pab-1* is required throughout postembryonic germline development. (A1-E3) *pab-1* RNAi was administered at each larval stage for 24 hours. Germline development was observed when RNAi-treated worms reached the adult stage by co-immunostaining with anti-PGL-1, a germline-specific marker, and monoclonal antibody SP56, which specifically recognizes sperm, along with TO-PRO-3 nuclear staining. Dissected gonads from adult animals treated with *pab-1* RNAi when they were at the L1 (B1-B3), L2 (C1-C3), L3 (D1-D3), and L4 (E1-E3) stages are shown. A control adult gonad with mock RNAi treatment (A1-A3) is also shown. Dissected gonads (outlined by dashed lines) are oriented with the distal ends at the right side. Average numbers of germ cells per gonad arm after each RNAi treatment are shown at the bottom of each DNA image. Asterisk indicates the distal end of each gonad. Circles in A2, D2, and E2 indicate SP56 signal. Bars, 10 µm. Localization of P granules after *pab-1* RNAi treatment (G1-G3) is shown along with mock RNAi control (F1-F3). (H) Average number of germ cells per gonad arm along with the expression level of *pab-1* mRNA measured by quantitative real-time RT-PCR at embryonic, L1, L2-L3, L4 larval, and young adult (yA) stages in wild-type N2 are shown. Error bars represent s.d. (I) Western blot analysis of PAB-1 protein expression with anti-PAB-1, in *pab-1* RNAi-treated and control *rrf-1*(pk1417) animals, as well as in wild-type N2, *pab-1*(bn116) and *pab-1*(bn119) mutants, and in the germline proliferation defective glp-1(q231) mutant. α-Tubulin was used as a loading control.

In the previous study, we demonstrated that *pab-1* mutants were defective in germline stem cell proliferation [22]. To understand further the functions of PAB-1 during germline development, we treated the *rrf-1*(*pk1417*) mutant, in which RNAi is effective only in the germline and limited somatic tissues [34], with *pab-1* dsRNA at each larval stage for 24 hours. The *pab-1* RNAi-treated worms were then recovered to seeded NGM plates, allowed to develop to adult stage, and their germline was observed after double immunostaining with anti-PGL-1, a germline-specific P-granule marker (Figure 1A1, B1, C1, D1, E1), and SP56 [35], a sperm marker (Figure 1A2, B2, C2, D2, E2), along with TO-PRO-3 nuclear staining (Figure 1A3, B3, C3, D3, E3). *pab-1* RNAi treatment at the L1 stage resulted in germline proliferation defects, as observed in *pab-1* mutants (Figure 1B1–B3). We found that these germ cells were arrested before entering meiosis because they were negative for immunostaining with anti-HIM-3, a meiotic marker (Figure S1A–F) [36], indicating that *pab-1* RNAi phenotype is different from that of *glp-1*(*lf*) mutants, which exhibit premature meiotic entry without mitotic proliferation [37]. When *pab-1* RNAi was treated at the L2 stage, germ cells of the worms were moderately proliferated and entered meiosis (Figure S1G–I), but sperm were not produced (Figure 1C1–C3). When worms were treated with *pab-1* RNAi at the L3 stage, the worms produced sperm but not oocytes (Figure 1D1–D3). These worms, which were *pab-1* RNAi treated at the L1, L2, or L3 stage, all became sterile adults. *pab-1* RNAi at the L4 stage resulted in defective oogenesis (Figure 1E1–E3), as well as severely reduced brood size (17.1 ± 2.6, n=15) and high embryonic lethality of their progeny (50.8%, n=15). Moreover, the hatched embryos became sterile adults (data not shown). These results indicate that PAB-1 is required throughout the postembryonic germline development. This conclusion was further supported by the following observations: First, the expression level of *pab-1* mRNA increased as the number of germ cells, which was scored as the number of PGL-1-positive nuclei in the gonad, increased during the larval development in wild-type N2 (Figure 1H). Second, the level of PAB-1 protein, which was detected by our original rabbit polyclonal anti-PAB-1 antibody (see Materials and Methods), was drastically decreased in the *glp-1*(*q231*) mutant, which contained few germ cells, compared to wild-type N2 (Figure 1I). *pab-1* mRNA level was also significantly decreased in the *glp-1*(*q231*) mutant (Figure S2). Given that PAB-1 was still weakly detected in *glp-1*(*q231*) mutant, PAB-1 is likely enriched in but not specific to the germline. We also observed that after *pab-1* RNAi treatment, P granules were diffused (Figure 1G1–G3). This observation suggests that PAB-1 affects P granule integrity or assembly. The effectiveness of *pab-1* RNAi depletion in *rrf-1*(*pk1417*) mutant was confirmed by western blot analysis of the *rrf-1*(*pk1417*) mutant total worm proteins with or without *pab-1* RNAi treatment using anti-PAB-1 antibody, and the specificity of the anti-PAB-1 antibody was demonstrated using *pab-1* mutant alleles, *bn116* and *bn119* (Figure 1I). 

### PAB-1 Localizes to P Granules and the Cytoplasm in the germline and Embryos

**Figure 2:**
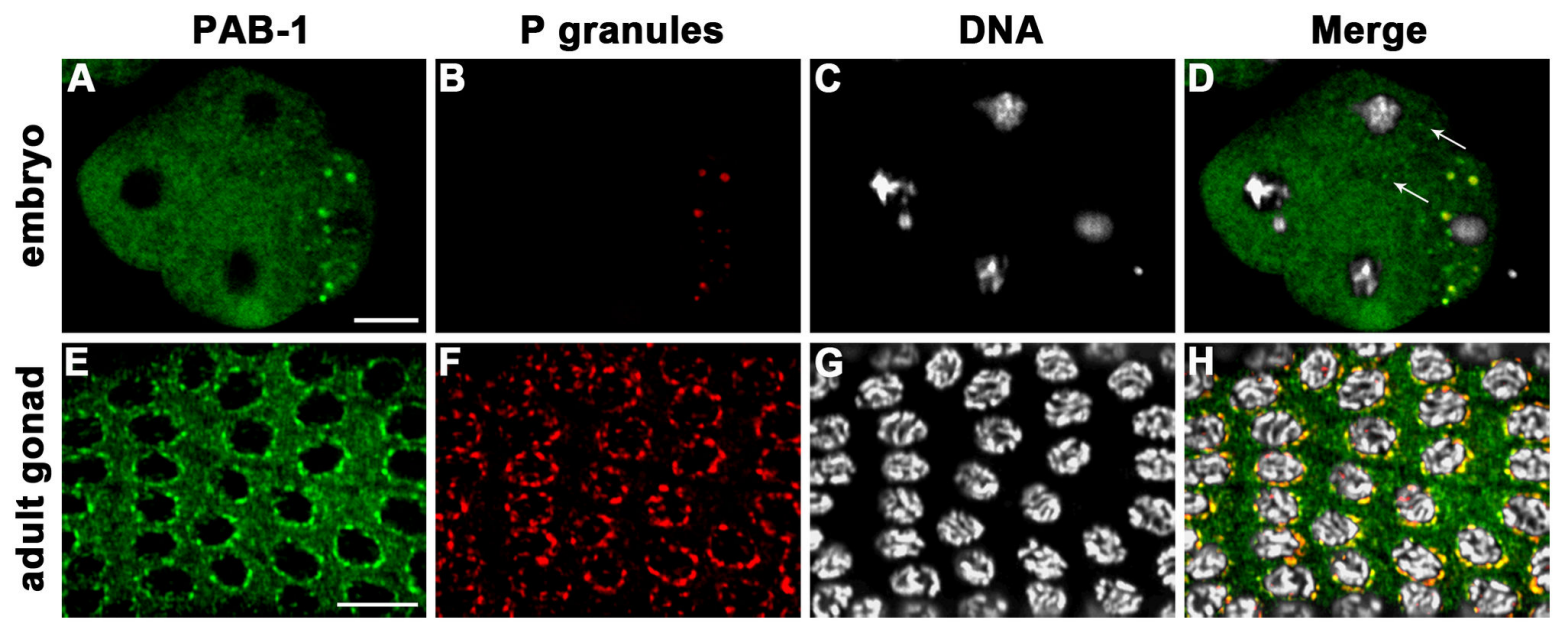
PAB-1 localizes to P granules and the cytoplasm in the embryos and adult gonads. (A–H) Localization of PAB-1 in an embryo and in an extruded adult gonad of wild-type N2 was observed by co-immunostaining with anti-PAB-1 (A, E) and OIC1D4, a P-granule marker (B, F), along with TO-PRO-3 for nuclear staining (C, G). Merged images are shown (D, H). The embryo is oriented such that the anterior side is on the left. Arrows in (D) indicate PAB-1 granules that are not colocalized with P granules. Bars, 10 µm.

To determine the localization of PAB-1, embryos and adult gonads of wild-type N2 were immunostained with anti-PAB-1 (Figure 2). PAB-1 localized to P granules and it was also dispersed in the cytoplasm in embryos (Figure 2A–D) and extruded adult gonads (Figure 2E–H). Localization of PAB-1 to P granules was demonstrated by co-immunostaining with OIC1D4 [38], a monoclonal antibody that recognizes P granules (Figure 2B, F), along with Hoechst 33342 nuclear staining (Figure 2C, G). PAB-1 granules that were not positive by OIC1D4 immunostaining were also observed in some somatic blastomeres in the embryos (Figure 2D, arrows). This expression pattern was also observed for some P-body components [15], suggesting that PAB-1 is a component of P-bodies.

**Figure 3:**
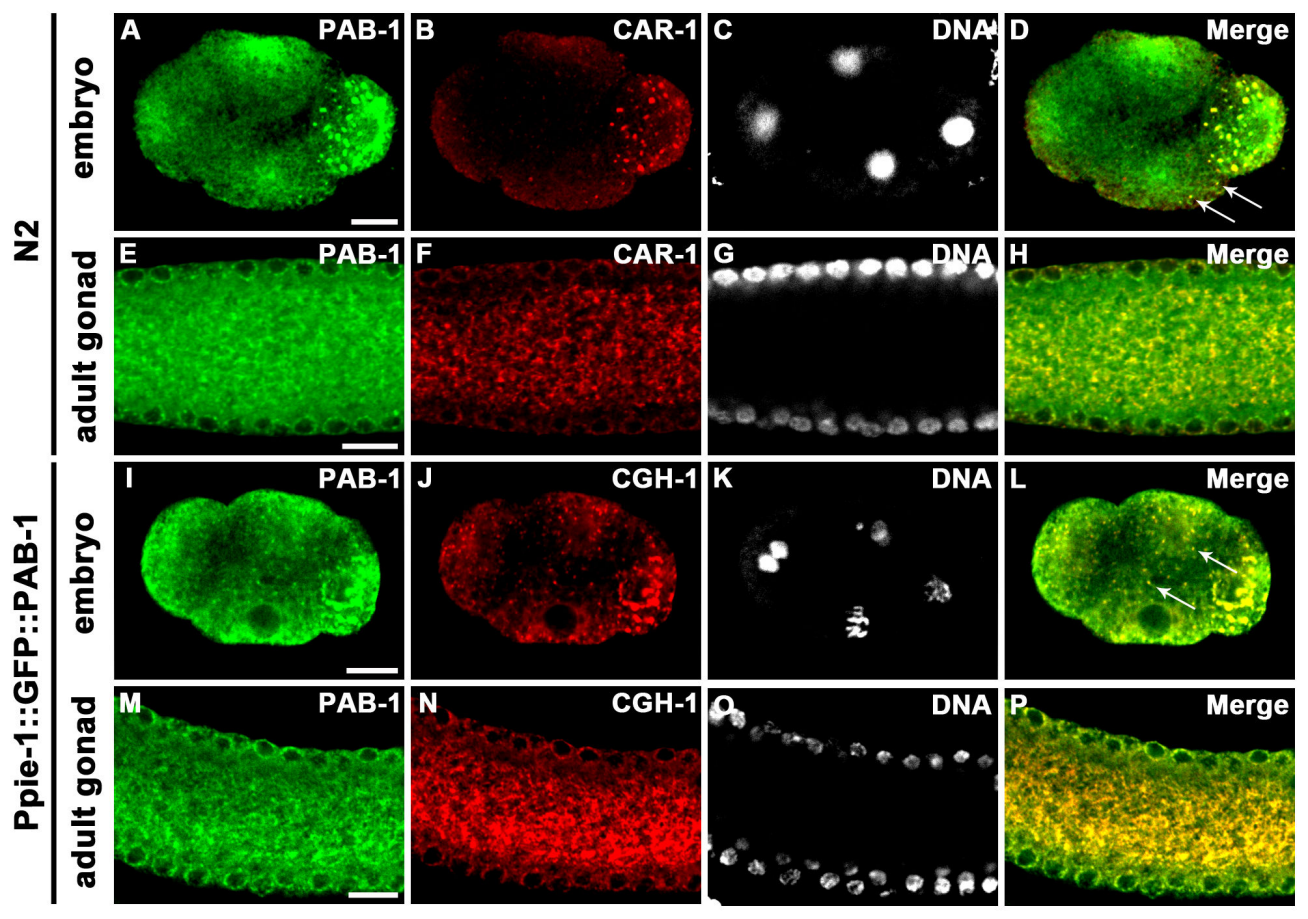
PAB-1 colocalizes with P-body components, CAR-1 and CGH-1, in embryos and adult gonads. (A–P) PAB-1 localization was analyzed by co-immunostaining of embryos and gonads with anti-PAB-1 along with anti-CAR-1 and anti-CGH-1. An embryo and an extruded adult gonad from wild-type N2 were co-immunostained with anti-PAB-1 (A, E) and anti-CAR-1 (B, F), along with nuclear staining (C, G). An embryo and an extruded adult gonad from a PAB-1::GFP transgenic strain were co-immunostained with anti-GFP (I, M) and anti-CGH-1 (J, N), along with nuclear staining (K, O). Merged images of the embryos (D, L) and the extruded adult gonads (H, P) are also shown. Arrows indicate colocalization of PAB-1 with CAR-1 (D), and PAB-1 with CGH-1 (L) in the somatic blastomeres. Bars, 10 µm.

To determine whether PAB-1 colocalizes with other P-body components, embryos and adult gonads of N2 were co-immunostained with anti-PAB-1 and anti-CAR-1 [18], and embryos and adult gonads of transgenic worms expressing GFP::PAB-1 under the control of *pie-1* promoter (a gift from A. Sugimoto) were co-immunostained with anti-GFP and anti-CGH-1 [19] (Figure 3). CAR-1 and CGH-1 are major components of P-bodies [18,20,39,40]. We found that PAB-1 colocalized with CAR-1 (Figure 3A–H) and CGH-1 (Figure 3I–P) in P granules and rachis in the embryos and gonads. Moreover, PAB-1 partially colocalized with CAR-1 (Figure 3D, arrows) and CGH-1 (Figure 3L, arrows) as foci in some somatic blastomeres in the embryos. These observations suggest that PAB-1 is a component of P-bodies. 

### PAB-1 affects the localization of CGH-1 and CAR-1

**Figure 4:**
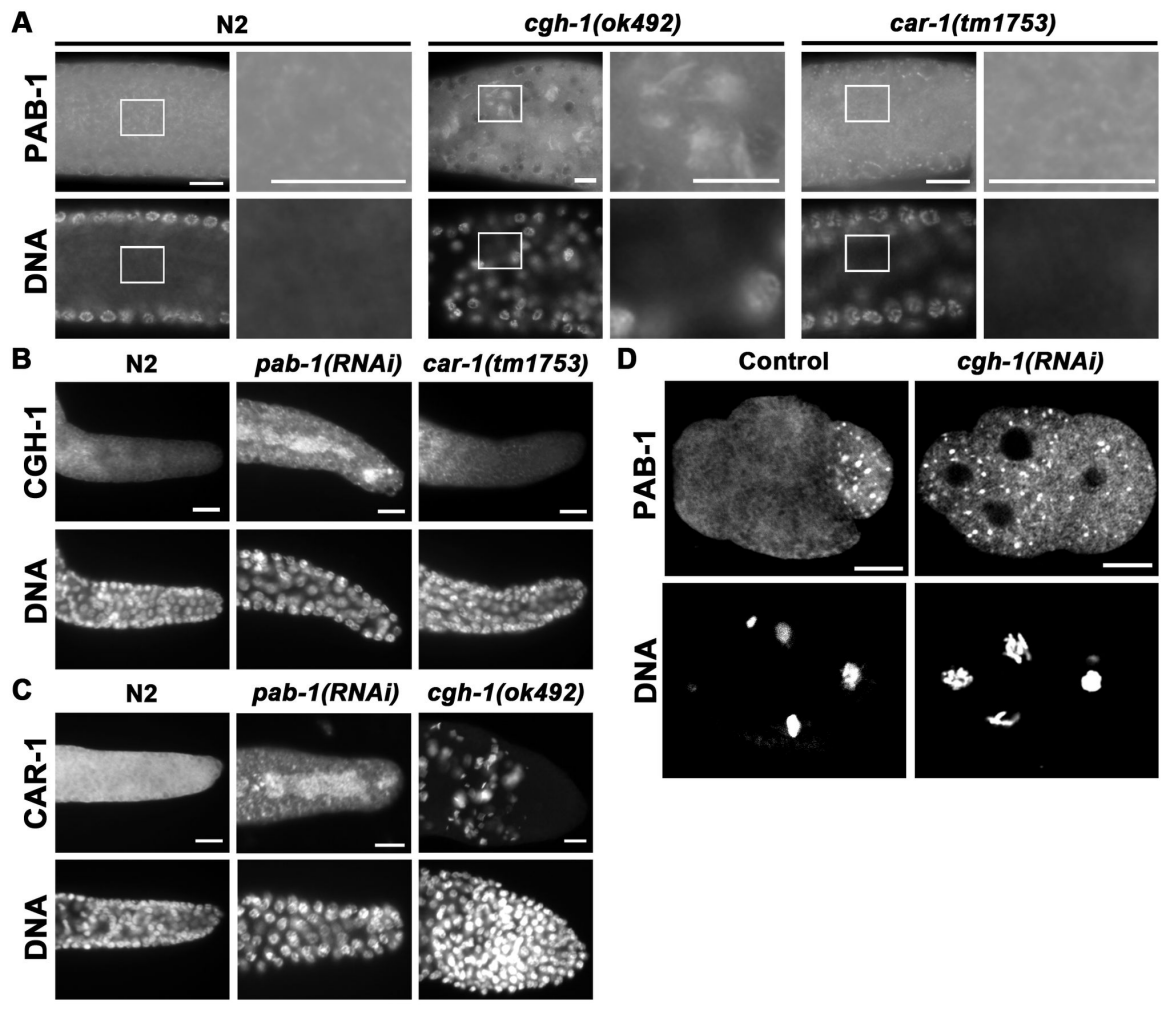
PAB-1 and CGH-1 mutually affect the other’s localization. (A–C) Extruded gonads from wild-type N2, *cgh-1*(ok492), car-*1*(tm1753), and *pab-1*(RNAi) adult hermaphrodites were immunostained with anti-PAB-1 (A), anti-CGH-1 (B), or anti-CAR-1 (C) as indicated, along with nuclear staining. (A) Enlarged images of the indicated regions are shown at the right of each original image. Dissected gonads are oriented such that the distal ends are at the right (A–C). (D) Embryos from *cgh-1* RNAi-treated and control animals were immunostained with anti-PAB-1, along with nuclear staining. The control embryo is oriented with the anterior side at the left. The orientation of the *cgh-1* RNAi-treated embryo could not be determined. Bars, 10 µm.

Having observed that PAB-1 colocalized with CAR-1 and CGH-1, to further explore the interaction among them, we examined the localization of each protein in the absence of other P-body components by immunostaining (Figure 4). PAB-1 localized to P granules around the germ nuclei, and it was also evenly dispersed in the rachis in adult gonads of wild-type N2 (Figure 4A). However in *cgh-1*(*ok492*) mutants, the localization of PAB-1 was significantly affected (Figure 4A). PAB-1 was aggregated as patched structures in the rachis of *cgh-1*(*ok492*) gonads. The PAB-1 aggregates colocalized with CAR-1 in the *cgh-1*(*ok492*) gonads (data not shown). In contrast to *cgh-1*(*ok492*) mutant, localization of PAB-1 was not affected in car-*1*(*tm1753*) gonads (Figure 4A). These results suggest that the localization of PAB-1 is influenced by CGH-1 but not by CAR-1. Altered localization of PAB-1 was also observed in embryos after *cgh-1* RNAi (Figure 4D). PAB-1 foci were concentrated mainly in the P_2_ blastomere in the control (mock RNAi) 4-cell embryo, whereas in the *cgh-1*(*RNAi*) 4-cell embryo, they were dispersed to all the blastomeres (Figure 4D). 

To analyze whether the absence of PAB-1 affects the localization of CGH-1 or CAR-1, we examined the localization of CGH-1 and CAR-1 in *pab-1*(*RNAi*) gonads by immunostaining with anti-CGH-1 (Figure 4B) and anti-CAR-1 (Figure 4C). CGH-1 and CAR-1 were localized to perinuclear P granules and were also dispersed in the rachis in N2 gonads. However, in *pab-1*(*RNAi*) gonads, CGH-1 and CAR-1 accumulated in the rachis (Figure 4B, C). On the other hand, while the localization of CGH-1 was not affected in car-*1*(*tm1753*) gonads (Figure 4B), CAR-1 localization was significantly affected in *cgh-1*(*ok492*) gonads (Figure 4C). That is, CAR-1 formed large sheet-like structures in *cgh-1*(*ok492*) gonads, as previously reported [18,20]. These results indicate that PAB-1 and CGH-1 mutually affect the other’s localization. In contrast, although PAB-1 and CGH-1 affect the localization of CAR-1, CAR-1 does not affect the localization of PAB-1 or CGH-1. 

### Depletion of *pab-1* decreased the mRNA levels of germline-enriched genes

**Figure 5:**
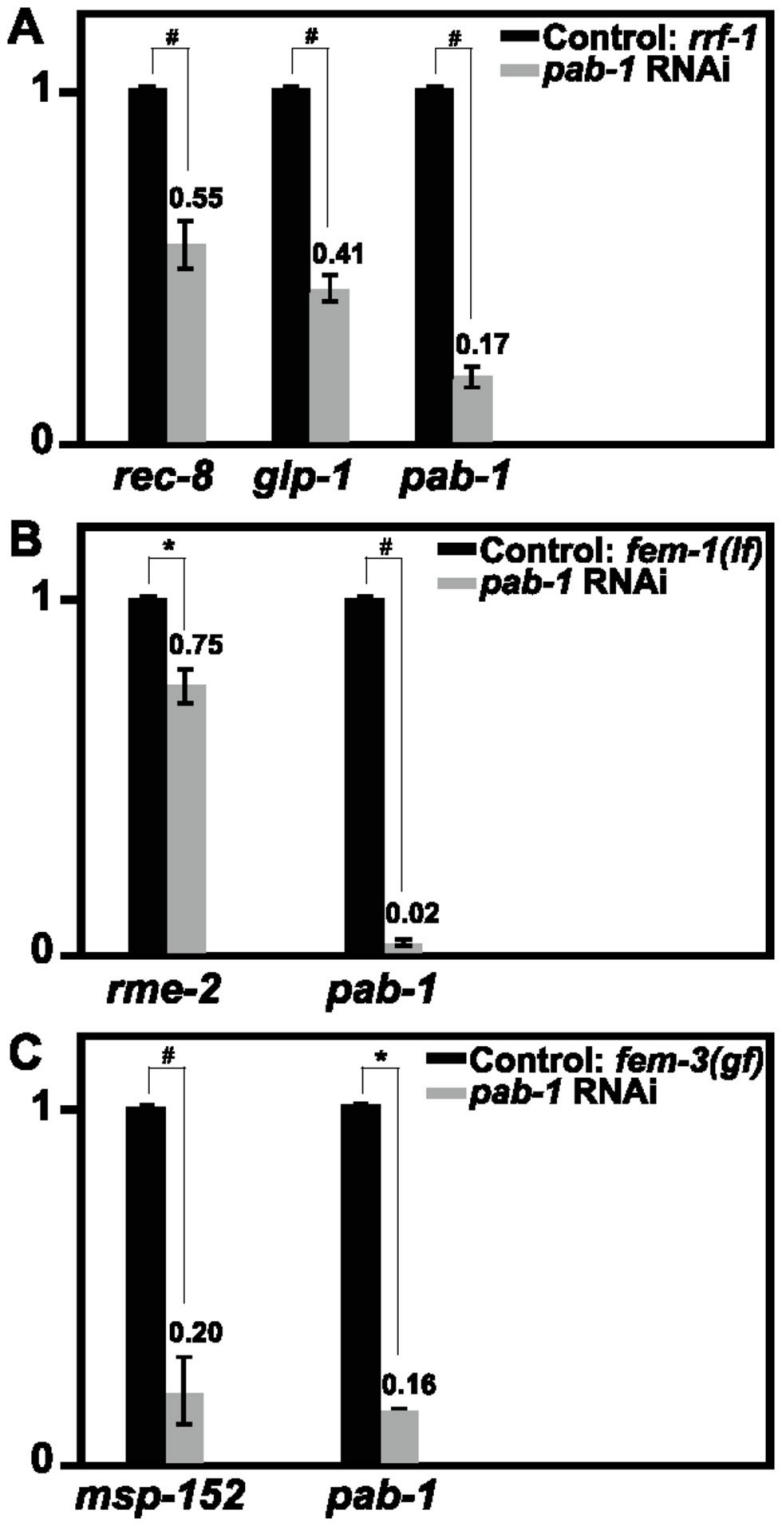
*pab-1* RNAi reduced the mRNA levels of germline-enriched genes. Relative mRNA expression levels with or without *pab-1* RNAi of *rec-8*, glp-1, and *pab-1* in *rrf-1*(pk1417) (A); *rme-2* and *pab-1* in fem*-*1(hc17) (B); and *msp-152* and *pab-1* in fem*-*3(q20) (C) are shown. * *p* < 0.05, ^#^ *p* < 0.005. The average values from 3 independent experiments were normalized to that of act*-*1, and the relative expression levels are shown with the control values taken as 1. Error bars represent s.d.

**Figure 6:**
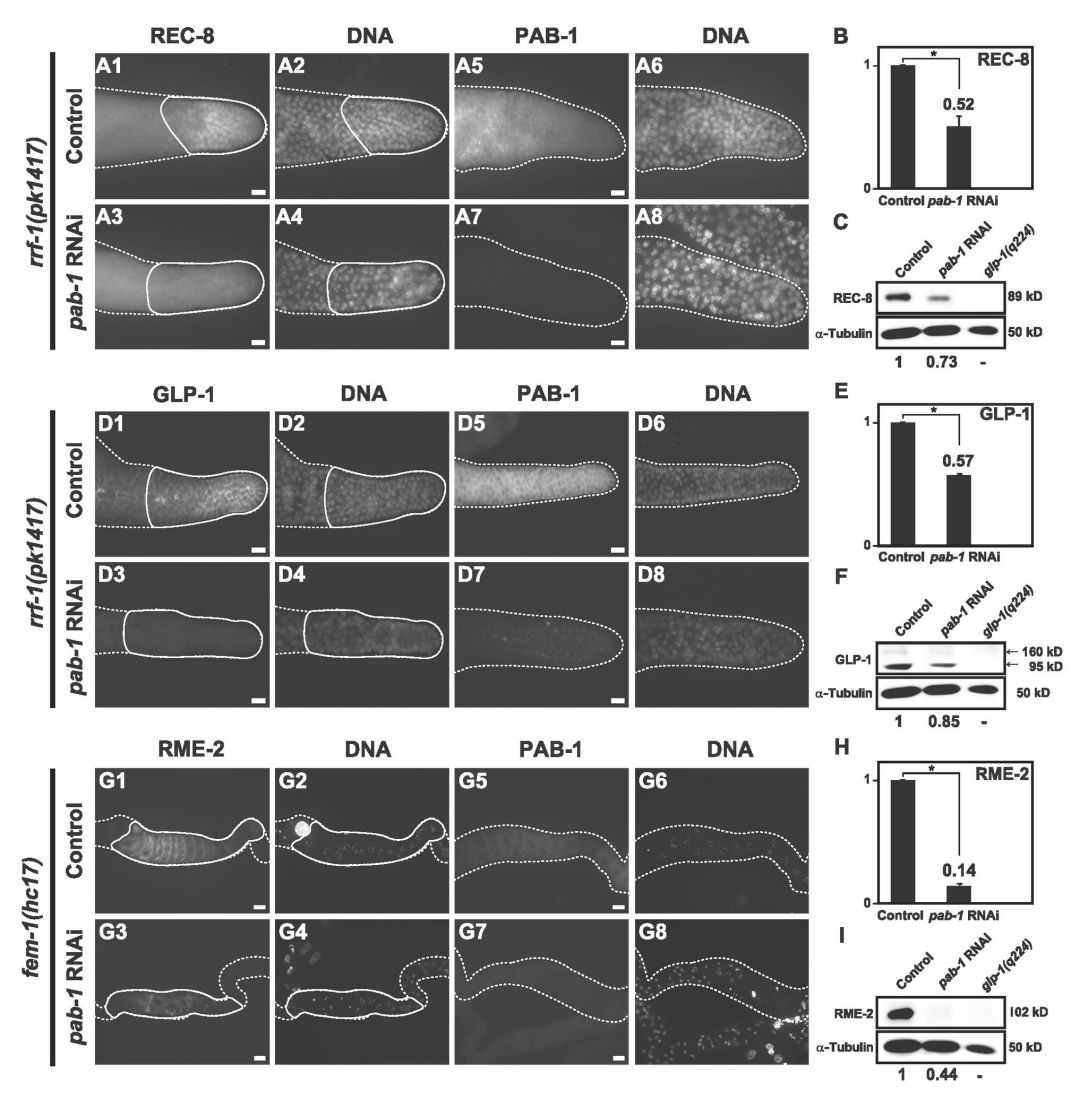
*pab-1* RNAi reduced the protein levels of germline-enriched genes. The protein expression of REC-8 and GLP-1 in *rrf-1*(pk1417) worms as well as RME-2 in fem*-*1(hc17) worms, with or without *pab-1* RNAi, was analyzed by immunostaining and western blotting. Extruded gonads of RNAi-treated and control animals were immunostained with anti-REC-8 (A1, A3), anti-GLP-1 (D1, D3), anti-RME-2 (G1, G3), and anti-PAB-1 (A5, A7, D5, D7, G5, G7), along with nuclear staining (A2, A4, D2, D4, G2, G4, A6, A8, D6, D8, G6, G8). Dotted lines indicate the borders of gonads. The extruded gonads are oriented such that the distal ends are on the right. Bars, 10 µm. (B, E, H) The protein level within the region enclosed with a line in each panel was digitally quantified. The relative protein level is shown with the control values taken as 1. (C, F, I) Western blot analyses of REC-8, GLP-1, and RME-2 with or without *pab-1* RNAi are shown. Protein extract from glp-1(q224), a germline proliferation defective mutant, was used as a negative control. α-Tubulin was used as a loading control. (F) The antibody against the intracellular region of GLP-1 recognized 160 kDa and 95 kDa protein bands as indicated [27]. *P* values were calculated by Student’s *t*-test. * *p* < 0.05.

To determine whether PAB-1 plays a role in mRNA stability in the germline, we measured mRNA levels of representative germline-enriched genes by quantitative real-time RT-PCR. *rec-8* and *glp-1*, which are strongly expressed in mitotic germ cells, and *rme-2* and *msp-152*, which are specifically expressed during oogenesis and spermatogenesis, respectively, were examined with or without *pab-1* RNAi, and their relative mRNA levels were shown after normalization to *act-1* (Figure 5). *act-1* was used as an internal control because its mRNA level was not significantly changed with or without *pab-1* RNAi (data not shown). The effectiveness of *pab-1* RNAi depletion was confirmed by simultaneously measuring the mRNA level of *pab-1* after *pab-1* RNAi in each set of experiments (Figure 5). *pab-1* RNAi was treated for 24 hours to the L4-stage *rrf-1*(*pk1417*) hermaphrodites, which already contained well proliferated mitotic germ cells. Therefore, numbers of germ cells were not significantly decreased by this RNAi treatment. Further, the RNAi treated worms were recovered to seeded NGM plates and cultured for 2 more days before harvesting them as adult worms. We found that after *pab-1* RNAi treatment, *rec-8* and *glp-1* mRNA levels were reduced to 55% and 41%, respectively, of the mock RNAi control levels (Figure 5A). *rme-2* mRNA level was reduced to 75% of the mock RNAi control level after *pab-1* RNAi in feminized *fem-1*(*lf*) worms, which produce only oocytes (Figure 5B). *msp-152* mRNA level was reduced to 20% of the mock RNAi control level after *pab-1* RNAi in masculinized *fem-3*(*gf*) worms, which produce only sperm (Figure 5C). These results indicate that RNAi depletion of *pab-1* at the L4 stage significantly decreased the mRNA levels of multiple germline-enriched genes without significantly affecting the numbers of germ cells that expressed these genes. This finding was further verified by measuring the protein levels of REC-8, GLP-1, and RME-2 with or without *pab-1* RNAi at the L4 stage either by digitally quantifying immunostaining signals (Figure 6A, B, D, E, G, H) or by western blot analysis (Figure 6C, F, I). The results of immunostaining signal quantification revealed that protein levels of REC-8, GLP-1, and RME-2 were reduced to 52%, 57%, and 14%, respectively, of the mock RNAi control levels after *pab-1* RNAi (Figure 6B, E, H). The results of western blot analysis showed that protein levels of REC-8, GLP-1, and RME-2 were reduced to 73%, 85%, and 44%, respectively, of the mock RNAi control levels after *pab-1* RNAi (Figure 6C, F, I). These results confirmed that protein levels of germline-enriched genes were reduced along with their mRNA levels after *pab-1* RNAi depletion. 

### PAB-1 is required for the maintenance of germline-enriched mRNAs

**Figure 7:**
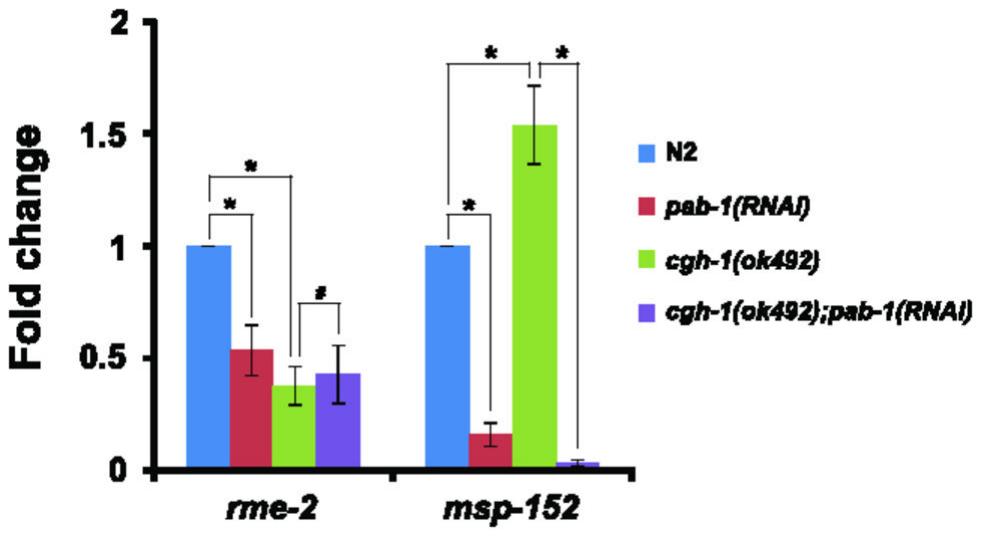
*pab-1* RNAi reduced the increased mRNA level of *msp-152* in the *cgh-1* mutant. Relative mRNA expression levels of *rme-2* and *msp-152* in N2, *pab-1* RNAi treated N2, *cgh-1*(ok492), and *pab-1* RNAi treated *cgh-1*(ok492) are shown. The average values from 3 independent experiments were normalized to that of act*-*1, and the relative expression levels are shown with the N2 values taken as 1. *P* values were calculated by Student’s *t*-test. *p < 0.001, ^#^ *p* = 0.160069. Error bars represent the s.d.

**Figure 8:**
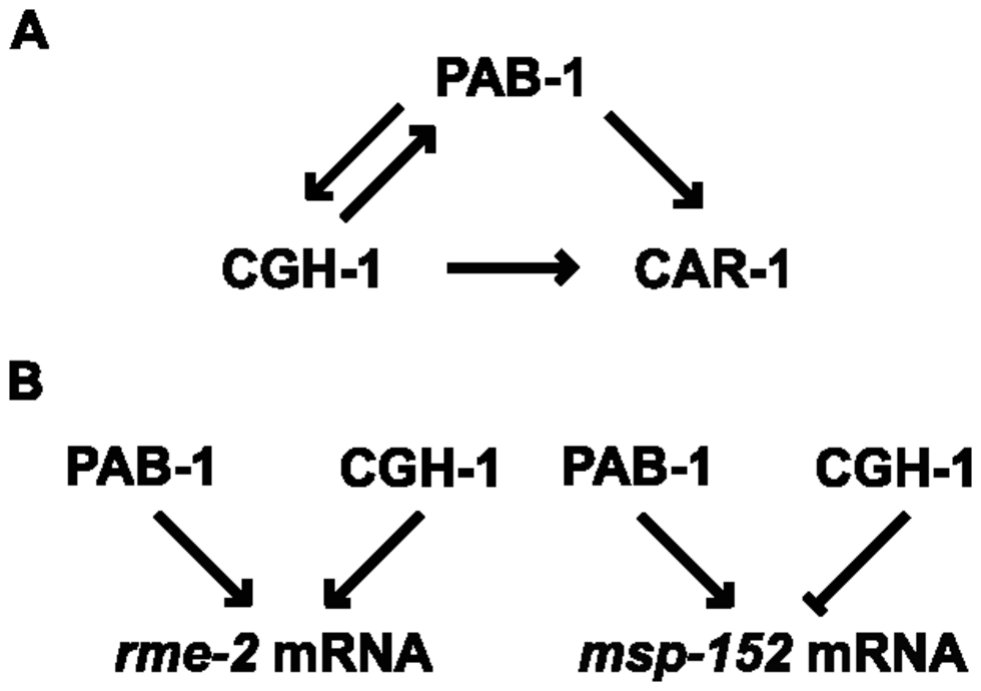
A proposed model for the function of PAB-1 together with CGH-1 and CAR-1. (A) Regulation of localization: During the assembly of P-bodies, PAB-1 and CGH-1 mutually affect the other’s localization. PAB-1 and CGH-1 affect CAR-1 localization, but CAR-1 does not affect the localization of PAB-1 or CGH-1. Arrows indicate that a downstream protein’s localization is affected by the absence of an upstream protein. (B) Regulation of mRNA metabolism: To regulate germline-enriched mRNAs, PAB-1 always functions positively for the accumulation of mRNAs, whereas CGH-1 functions either positively or negatively for the maintenance of mRNAs. In case of *msp-152*, PAB-1 and CGH-1 counteract each other to regulate the mRNA level. Arrows indicate positive regulation of mRNAs, including stabilization, accumulation, or protection. The T bar indicates negative regulation of mRNAs, including degradation.

CGH-1 is required for the accumulation of a subset of germline-enriched mRNAs in *C. elegans* [33,41,42]. PAB-1 and CGH-1 mutually affected the other’s localization, and depletion of *pab-1* decreased the mRNA levels of several germline-enriched genes in our study. Therefore, we examined the functional relationship between PAB-1 and CGH-1 in mRNA metabolism by measuring the mRNA levels of representative germline-enriched genes in the *cgh-1*(*ok492*) mutant with or without *pab-1* RNAi. The mRNA level of *rme-2* was reduced in *cgh-1* mutant (Figure 7), as previously reported [33]. In contrast, the mRNA level of *msp-152* was increased 1.5-fold in the *cgh-1* mutant compared to wild-type N2 (Figure 7). We then investigated whether RNAi depletion of *pab-1* can reduce the high level of *msp-152* mRNA in the *cgh-1* mutant. The mRNA level of *msp-152* was indeed reduced to 2.1% of the mock RNAi control level after *pab-1* RNAi in the *cgh-1* mutant (Figure 7). On the other hand, the mRNA level of *rme-2* was not significantly changed after *pab-1* RNAi in the *cgh-1* mutant, possibly because the mRNA level of *rme-2* was already reduced in the *cgh-1* mutant, and *pab-1* RNAi treatment, therefore, could not further reduce the mRNA level of *rme-2* in the *cgh-1* mutant. These results suggest the following. First, PAB-1 is required for the accumulation or stabilization of a subset of germline-enriched mRNAs including *msp-152*. Second, CGH-1 may function not only towards the accumulation of mRNAs but also aid in the degradation of a subset of germline-enriched mRNAs including *msp-152*. Therefore, PAB-1 and CGH-1 may counteract each other to regulate *msp-152* mRNA stability (Figure 8). 

## Discussion

Previous studies carried out by us as well as by others showed that *pab-1* mutants exhibit a specific defect in germline stem cell proliferation [21–23]. In this study, we demonstrated that *pab-1* RNAi treatment at each larval stage resulted in the arrest of germline development at the following stage (Figure 1). These results indicate that *pab-1* has essential functions not only for germline stem cell proliferation but also for later stages of germline development. These functions, masked in the studies of *pab-1* mutants with proliferation defects, have been revealed for the first time in this study by a series of stage-specific *pab-1* RNAi treatments. 

There are 2 cytoplasmic PABPs, PAB-1 and PAB-2, in *C. elegans*. The *pab-2* gene is located on the X chromosome; thus, germline specific inactivation of the X chromosome prevents its expression in the germline [23]. On the other hand, PAB-1 protein and *pab-1* mRNA was weakly detected in the *glp-1*(*q231*) mutant, which contains few germ cells (Figure 1), indicating that PAB-1 is germline-enriched but is also expressed weakly in the soma. Due to the soma-limited *pab-2* activity, *pab-1* mutants or *pab-1* RNAi would show germline-specific defects without causing any somatic defects. Nevertheless, the *pab-1*(*ok1656*) deletion null mutant showed larval arrest (data not shown). This result indicates that depletion of *pab-1* activity can cause somatic defects under certain conditions. To suppress possible somatic defects, we used *rrf-1* mutants for *pab-1* RNAi analysis in this study. In *rrf-1* mutants, RNAi is effective only in the germline and limited somatic tissues [34,43]. 

PAB-1 was shown to colocalize with CGH-1 and CAR-1, which are RNA-binding proteins and components of P-bodies (Figure 3). P-bodies are cytoplasmic aggregates consisting of RNAs and proteins, which include translational repressors and components of the mRNA decay machinery [13,14]. Among the components of P-bodies, a DEAD-box RNA helicase, Dhh1/Me31B/RCK/p54, is involved in the translational repression and decapping of mRNAs, and CGH-1 is a *C. elegans* ortholog of Dhh1 [19,44,45]. CGH-1 promotes the stability of numerous maternal mRNAs in some cases [33,41,42]. On the other hand, CAR-1, an ortholog of Scd6/*trailer hitch*/RAP55 containing Sm-like and FDF domains represses translation of target mRNAs [46–48]. Colocalization of PAB-1 with CGH-1 and CAR-1 suggests functional interactions among them. Our finding that PAB-1, CGH-1, and CAR-1 mutually affect each other’s localization further supports this view (Figure 4). In previous reports, PAB-1 and CAR-1 were found in CGH-1 immunoprecipitates [18,41]. However, CGH-1 was absent after RNase treatment in CAR-1 immunoprecipitates [20]. These observations suggest that interactions among PAB-1, CGH-1, and CAR-1 are RNA-mediated. In *pab-1* depleted conditions, CGH-1 and CAR-1 formed aggregates in the rachis (Figure 4B-C). To test the possibility that generation of these aggregates is a consequence of general translational arrest, rather than because they require PAB-1 for their proper localization, we observed CGH-1 and CAR-1 localization after RNAi treatment of *ifg-1*, which encodes the sole *C. elegans* ortholog of eIF4G, an essential component of translation initiation complex (Figure S3G–I) [49]. After *ifg-1*RNAi treatment, CGH-1 and CAR-1 also formed minor aggregates, but the degree of accumulation of CGH-1 and CAR-1 in the rachis was not so prominent compared to that in *pab-1*(*RNAi*) gonads (Figure S3D-F). From these observations we consider that aggregation of CGH-1 and CAR-1 after *pab-1* depletion is not a simple consequence of a translational arrest. 

RNAi depletion of *pab-1* decreased the mRNA levels of *rec-8*, *glp-1*, *rme-2*, and *msp-152*, 4 representative germline genes that function at different developmental stages (Figure 5). In addition, their protein levels were also reduced after *pab-1* RNAi (Figure 6). These results indicate that PAB-1 is required to maintain the mRNA and protein levels of multiple germline genes. This may be achieved by promoting mRNA stability or stimulating translational initiation, given that PABP is actively involved in these processes in yeast and mammals [50,51]. PAB-1 was enriched in the polysome fractions of total worm extract, suggesting that PAB-1 is actively involved in translation [33]. Among the 4 germline genes, the protein level of RME-2 was more significantly reduced (Figure 6H, I) than the mRNA level (Figure 5B) after *pab-1* RNAi compared to the untreated controls. This supports the view that PAB-1 regulates gene expression not only at the mRNA level but also at the translational level. 

In the *cgh-1* mutant, the mRNA level of *msp-152* was increased, whereas that of *rme-2* was decreased (Figure 7). However, the increased *msp-152* mRNA level in the *cgh-1* mutant was significantly reduced upon *pab-1* RNAi treatment as in wild-type N2 (Figure 7). This result indicates that PAB-1 functions consistently as a positive regulator for the stabilization or accumulation of mRNAs (Figure 8). Our study suggests that PAB-1, CGH-1, and CAR-1 regulate the mRNA levels of germline genes by associating and by functionally cooperating for the proper germline development. 

## Materials and Methods

### Worm culture and strains

*C. elegans* strains were cultured and handled as described previously [52]. The strains used were: N2 (Bristol wild-type strain), *pab-1*(*bn116*)*/hT2*[*bli-4(e937*)* let-?(q782*)* qIs48*] *(I;III*)*, pab-1(bn119*)*/hT2[bli-4(e937*)* let-?(q782*)* qIs48*]* (I;III*)*, rrf-1(pk1417*)*I, glp-1(q231*)*III, glp-1(q224*)*III, cgh-1(ok492*)*/hT2[bli-4(e937*)* let-?(q782*)* qIs48*]* (I;III*)*, car-1(tm1753*)*/hT2[bli-4(e937*)* let-?(q782*)* qIs48*] (*I*;III)*, fem-1(hc17*)*IV, fem-3(q20*)*IV*, and *Ppie-1::gfp::pab-1*. All strains were maintained at either 16°C or 20°C on Nematode Growth Medium (NGM) agar plates containing *Escherichia coli* strain OP50 and were occasionally grown at 25°C when necessary.

### dsRNA Interference (RNAi)

RNAi was performed by the soaking method with minor modifications [53]. The DNA template for *pab-1* dsRNA synthesis was amplified from N2 cDNA by PCR with a T7 promoter sequence using the following primers: 5′-GTAATACGACTCACTATAGGGCG-AAATGAACGTCGCT-3′ and 5′-GTAATACGACTCACTATAGGGCTTGCTTCTGAGCG-G-3′. In vitro transcription and purification of dsRNA were performed as described previously [54]. To examine the effects of *pab-1* depletion at each developmental stage, synchronized worms were soaked in dsRNA solution at each stage for 24 hours at 20°C, transferred to NGM plates seeded with OP 50, and allowed to develop to adults. Their germline development was then examined. Each set of experiment was repeated three times using 100 worms. To measure mRNA and protein levels after RNAi, L4 hermaphrodites (48 hours after synchronized L1) were treated with dsRNA for 24 hours. The treated worms were transferred to seeded plates and allowed to recover for 2 days. To measure mRNA levels after RNAi in *cgh-1*(*ok492*), young adult worms were treated. For *cgh-1* and *ifg-1* RNAi, dsRNA was transcribed in vitro from amplified cDNA templates flanked by T7 promoter sequences. The cDNA template was PCR-amplified from the yk85e1 and yk450c12 clone, respectively, using the primer pair 5′-GCGTAATACGACTCACTATAGGGAACAAAAGCTGGAGCT-3′ and 5′-GTAATACGACTCACTATAGGGC-3′. The yk cDNA clones were generously provided by Y. Kohara (National Institute of Genetics, Japan).

### Quantitative real-time RT-PCR

Total RNA was isolated from synchronized populations of L1, L2-L3, L4, and young adult worms of wild-type N2, from *glp-1*(*q231*) adult mutants, from *cgh-1*(*ok492*) adult mutants, and from *pab-1* RNAi-treated worms. Worms were collected in TRIzol (Invitrogen), and total RNA was extracted using a phase lock gel MaXtract High Density kit from Qiagen (Valencia, CA). cDNA was synthesized using an oligo-dT primer and M-MLV reverse transcriptase (Invitrogen). qPCR reactions were conducted using power SYBR® Green PCR Master Mix (Applied Biosystems) in a 96-well plate with a 25 µL reaction volume. Primers for *act-1*, which served as the internal control, were 5′-CCAGGAATTGCTGATCGTATGCAGAA-3′ and 5′-TGGAGAGGGAAGCGAGGATAGA-3′. Primers for *pab-1* were 5′-GTGCTAAGGT-CATGACTG-3′ and 5′-GTTGCGCTGCTGTT-3′. Primers for *rec-8* were 5′-TTTATGAGAA-CTGACGATCTGG-3′ and 5′-CCGATTATTGGGAAGGAGAC-3′. Primers for *glp-1* were 5′-CATCGACACCGAATCGAATGG-3′ and 5′-AGTTAGGAGATATGTTGGGAGG-3′. Primers for *rme-2* were 5′-ATGAAGACAATAAGTGTCGGAG-3′ and 5′-CGCTTGGAGC-ATTAGTTTGG-3′. Primers for *msp-152* were 5′-CAAGACCACCAATATGAAGAG-3′ and 5′-GTTGTTAGTGTCCTCCTGTC-3′. The relative mRNA expression level of each gene was averaged from triplicate measurements of 3 independent experiments and normalized to that of *act-1*, indicated as the control value as 1.

### PAB-1 antibody production

To generate antibodies against PAB-1, 186 nucleotides, corresponding to amino acid residues 409–470 of Y106G6H.2, were amplified from *pab-1* cDNA and inserted into pHIT198 (gift from Hiroaki Tabara, Tsukuba University, Japan) [55]. The purified 6X His-MBP fusion protein was outsourced for immunization of rabbits (Abfrontier). Anti-PAB-1 antiserum was affinity purified using GST-PAB-1 fusion protein-coupled Affigel-10 beads. 

### Immunofluorescence

Immunostaining was performed as previously described with minor variations [22,53]. Primary antibodies were used after diluting with PBS as follows: rabbit anti-PAB-1 (1:500), mouse monoclonal OIC1D4 (without dilution; DSHB, the University of Iowa), rabbit anti-PGL-1 (1:1000) [26], mouse monoclonal SP56 (without dilution) [35], rabbit anti-CGH-1 (1:100) [19], chicken anti-CAR-1 (1:100) [18], rabbit anti-REC-8 (1:500; NOVUS), rabbit anti-GLP-1 (1:5) [27], rabbit anti-RME-2 (1:50) [29], and mouse anti-GFP (1:100; Invitrogen), rabbit anti-HIM-3 (1:100) [36]. Secondary antibodies were used as follows: Alexa 488-conjugated goat anti-rabbit IgG (1:200; Molecular Probes), Alexa 546-conjugated goat anti-mouse IgG (1:200; Molecular Probes), and Alexa 546-conjugated goat anti-chicken IgG (1:200; Molecular Probes). All primary antibodies were incubated overnight and all secondary antibodies for 4 hours at 4°C. The specimens were counterstained for DNA with 0.5 µg/mL Hoechst 33342. Confocal images were acquired using a confocal microscope (FV-1000 spectral; Olympus) with FV10-ASW 2.0 software. Images were also acquired using a fluorescence microscope (Axioskop 2 MOT, ZEISS) and processed with Openlab software (Improvision). To quantify the expression levels of GLP-1, REC-8, and RME-2, specimens were imaged under identical exposure times and analyzed with Openlab. Images were processed using Photoshop CS5 and Illustrator CS5 (both from Adobe).

### Western blot analysis

For western blot analysis, up to 150 worms of each strain were collected in 20 µL sample buffer (10% glycerol + 60 mM Tris-HCl, pH 6.8, + 4% SDS + 0.05% bromophenol blue + 5% 2-mercaptoethanol) and then boiled at 100°C for 8 minutes. The protein samples were centrifuged at 13000 rpm for 10 minutes, and loaded onto 10% SDS-PAGE gels to detect PAB-1 and 7.5% SDS-PAGE gels to detect GLP-1, REC-8, RME-2, and MSPs and then transferred to PROTRAN membranes (Whatman) with a current of 60 mA for 75 minutes. Antibody dilutions were as follows: rabbit anti-PAB-1 (1:1000), rabbit anti-GLP-1 (1:5), rabbit anti-REC-1 (1:1000), rabbit anti-RME-2 (1:500), mouse 4A5 (1:10), mouse anti-α-tubulin (1:100; Sigma). After the addition of primary antibody, the membrane was incubated overnight at 4°C. Immunoreactive protein bands were detected with either HRP-conjugated goat anti-rabbit IgG (1:10,000; Santa Cruz Biotechnology) or HRP-conjugated donkey anti-mouse IgG (1:1000; Jackson ImmunoResearch). Blots were visualized with an ECL Plus kit (Amersham) and analyzed using LAS-3000 (Fuji Film). The relative abundance of GLP-1, REC-8, and RME-2 after *pab-1* RNAi was compared with controls, and the intensity of the bands was calculated by densitometry. α-Tubulin was used as a reference standard. 

## Supporting Information

Figure S1 ***pab-1* RNAi at the L1 stage causes developmental arrest of germ cells before entering meiosis.** Extruded gonads of *rrf-1*(pk1417) worms with or without *pab-1* RNAi treatment were co-immunostained with anti-HIM-3 (A, D, G), a meiotic marker, and OIC1D4 (B, E, H), a monoclonal antibody specifically recognizing P granules, along with TO-PRO-3 nuclear staining (C, F, I). *pab-1* RNAi was administered either at L1 (D–F) or L2 (G–I) stage for 24 hours. Germ cells were observed after RNAi-treated worms were recovered and grown to the adult stage. A control adult gonad arm with mock RNAi treatment (A–C) is also shown. Asterisk indicates the distal end of each gonad. Bars, 10 µm.
(TIFF)

Figure S2 ***pab-1* mRNA is decreased in the germline proliferation defective mutant.** The expression levels of *pab-1* mRNA measured by quantitative real-time RT-PCR in wild-type N2 and *glp-1*(*q231*) mutant are shown. The average values from 3 independent experiments were normalized to that of *act-1*, and the relative expression levels are shown with the N2 value taken as 1. *P* values were calculated by Student’s *t*-test. *p < 0.005. Error bars represent the s.d.
(TIFF)

Figure S3 **PAB-1 and IFG-1 differently affect localization of CGH-1 and CAR-1.** Extruded gonads of a mock RNAi treated control worm (A–C), a *pab-1* RNAi treated worm (D–F), and an *ifg-1* RNAi treated worm (G–I) were co-immunostained with anti-CGH-1 and anti-CAR-1 along with TO-PRO-3 nuclear staining. Asterisk indicates the distal end of each gonad. Bars, 10 µm.
(TIF)
