# Supplementary figures and images for: PAB-1, a Caenorhabditis elegans Poly(A)-Binding Protein, Regulates mRNA Metabolism in germline by Interacting with CGH-1 and CAR-1
Source: PLoS One. 2013 Dec 19;8(12):e84798. doi: 10.1371/journal.pone.0084798 (PMC3868610; doi:10.1371/journal.pone.0084798)

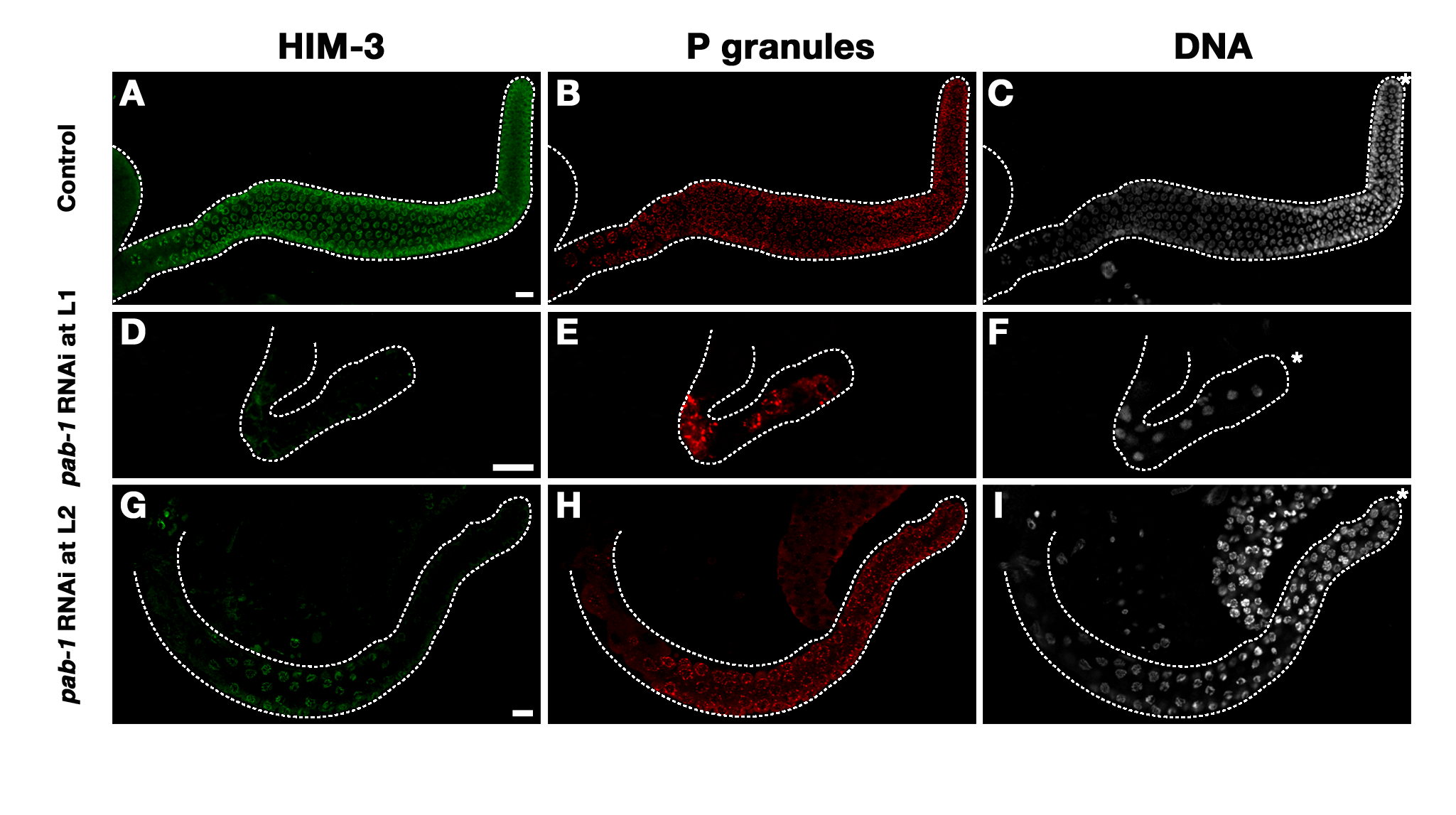

Supplement: Figure S1 — pab-1 RNAi at the L1 stage causes developmental arrest of germ cells before entering meiosis. Extruded gonads of rrf-1(pk1417) worms with or without pab-1 RNAi treatment were co-immunostained with anti-HIM-3 (A, D, G), a meiotic marker, and OIC1D4 (B, E, H), a monoclonal antibody specifically recognizing P granules, along with TO-PRO-3 nuclear staining (C, F, I). pab-1 RNAi was administered either at L1 (D–F) or L2 (G–I) stage for 24 hours. Germ cells were observed after RNAi-treated worms were recovered and grown to the adult stage. A control adult gonad arm with mock RNAi treatment (A–C) is also shown. Asterisk indicates the distal end of each gonad. Bars, 10 µm. (TIFF) [file pone.0084798.s001.tiff]

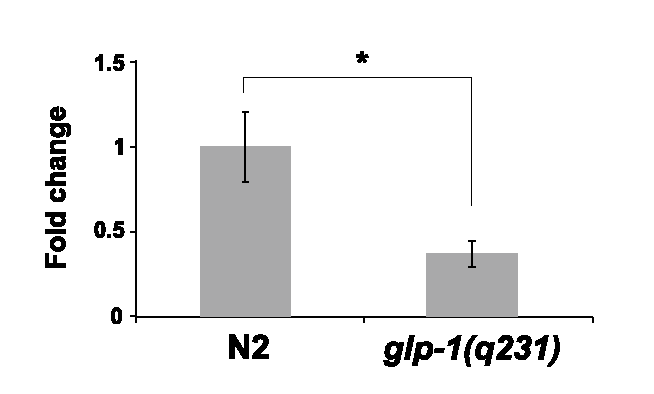

Supplement: Figure S2 — pab-1 mRNA is decreased in the germline proliferation defective mutant. The expression levels of pab-1 mRNA measured by quantitative real-time RT-PCR in wild-type N2 and glp-1(q231) mutant are shown. The average values from 3 independent experiments were normalized to that of act-1, and the relative expression levels are shown with the N2 value taken as 1. P values were calculated by Student’s t-test. *p < 0.005. Error bars represent the s.d. (TIFF) [file pone.0084798.s002.tiff]

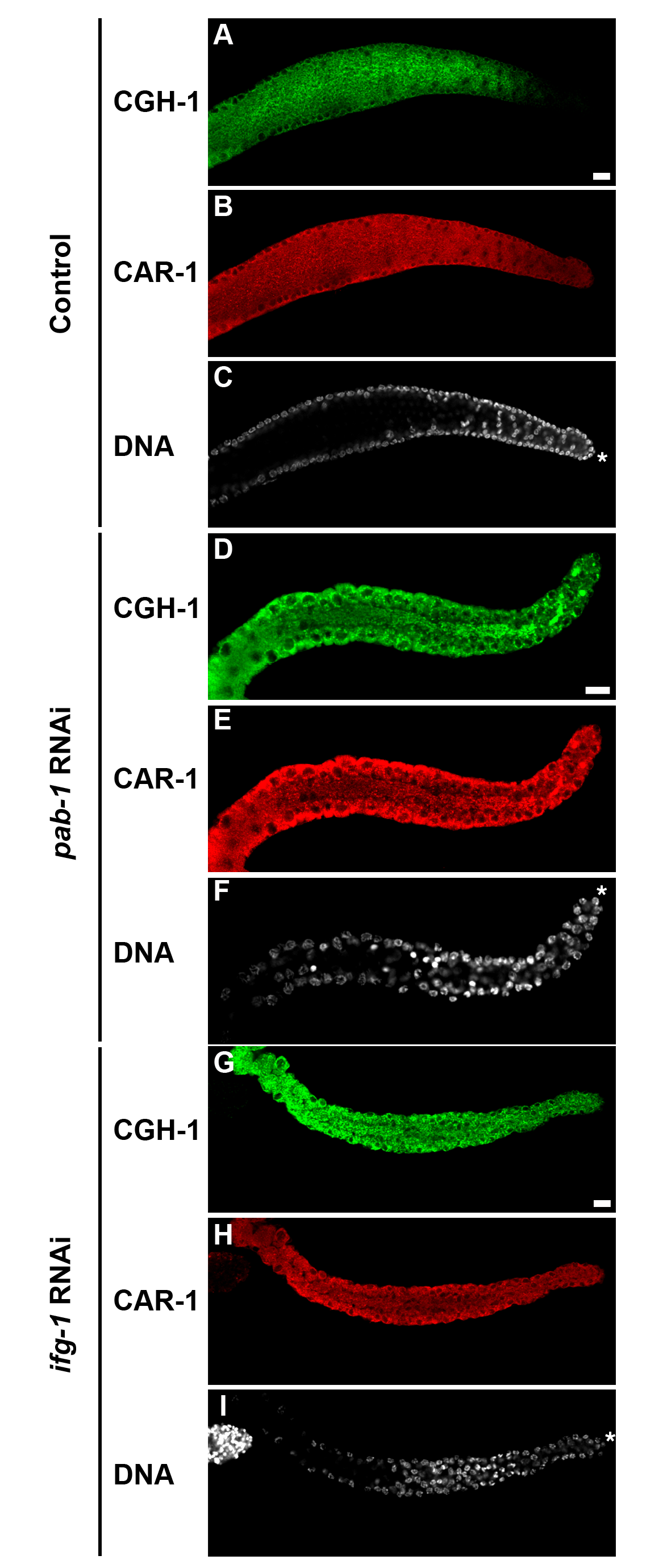

Supplement: Figure S3 — PAB-1 and IFG-1 differently affect localization of CGH-1 and CAR-1. Extruded gonads of a mock RNAi treated control worm (A–C), a pab-1 RNAi treated worm (D–F), and an ifg-1 RNAi treated worm (G–I) were co-immunostained with anti-CGH-1 and anti-CAR-1 along with TO-PRO-3 nuclear staining. Asterisk indicates the distal end of each gonad. Bars, 10 µm. (TIF) [file pone.0084798.s003.tif]
